# Physical Therapy in Neurorehabilitation with an Emphasis on Sports: A Bibliometric Analysis and Narrative Review

**DOI:** 10.3390/sports12100276

**Published:** 2024-10-12

**Authors:** George M. Pamboris, Spyridon Plakias, Anna Tsiakiri, Georgia Karakitsiou, Paschalina Bebeletsi, Konstantinos Vadikolias, Nikolaos Aggelousis, Dimitrios Tsiptsios, Foteini Christidi

**Affiliations:** 1 Department of Health Sciences, School of Sciences, European University Cyprus, Nicosia 2404, Cyprus; g.pamboris@euc.ac.cy; 2 Department of Physical Education and Sport Science, University of Thessaly, 42100 Trikala, Greece; spyros_plakias@yahoo.gr; 3 Neurology Department, School of Medicine, Democritus University of Thrace, 68100 Alexandroupolis, Greece; atsiakir@med.duth.gr (A.T.); bebeletsi@gmail.com (P.B.); kvadikol@med.duth.gr (K.V.); 4 Department of Psychiatry, Medical School, Democritus University of Thrace, 68100 Alexandroupolis, Greece; gkarakitsiou@yahoo.gr; 5 Department of Physical Education and Sport Science, Democritus University of Thrace, 69100 Komotini, Greece; nagelous@phyed.duth.gr; 6 3rd Department of Neurology, Aristotle University of Thessaloniki, 54124 Thessaloniki, Greece; tsiptsios.dimitrios@yahoo.gr

**Keywords:** neurological injuries, athlete rehabilitation, VOSviewer, performance analysis, science mapping, clustering

## Abstract

The increasing interest in physical therapy in sports neurorehabilitation stems from the high incidence of neurological injuries among athletes and the crucial role of rehabilitation in facilitating their safe return to sports. This study aims to provide a comprehensive analysis of research trends in physical therapy and neurorehabilitation in athletes. This study presents a bibliometric analysis of 103 documents from the Scopus database, followed by a narrative review of the identified thematic areas. Together, these approaches offer a comprehensive overview of the international literature on the application of physical therapy in sports neurorehabilitation, highlighting key trends and contributors. The software VOSviewer and Power BI (2.136.1202.0) were used for the bibliometric analysis and the visualization of the results. Techniques such as performance analysis (documents per year, top sources and countries in documents, and top authors in citations) and science mapping (co-authorship, bibliographic coupling, co-citation, and co-occurrence) were conducted. The results revealed the journals and the authors with the greatest impact in the field and collaborations between various countries. From the co-occurrence analysis of the keywords, three key thematic clusters were identified, Clinical Approaches and Outcomes in Neurorehabilitation, Athlete-Centered Neurorehabilitation Techniques, and Specialized Interventions in Sports Medicine and Neurorehabilitation, which were used to conduct the narrative review. These findings provide a solid foundation for future research and clinical practice aimed at enhancing recovery times and overall performance in athletes with neurological injuries.

## 1. Introduction

Physical therapy plays a crucial role in neurorehabilitation for athletes, aiding in recovery from injuries and enhancing performance. Neurological physical therapy plays a crucial role in assisting individuals with life-changing neurological conditions and injuries to improve their health and wellness despite their condition [1,2]. Due to the intense physical demands of their sports, athletes are at increased risk for various neurological injuries such as concussions, spinal cord injuries, and traumatic brain injuries [3,4,5,6]. These complex conditions can significantly affect an athlete’s physical, cognitive, and psychological well-being [7,8,9]. As a result, effective neurorehabilitation strategies are essential to ensure their safe return to sports and everyday activities [8,10].

Athletes are particularly vulnerable to neurological injuries such as concussions, traumatic brain injuries, and spinal cord injuries due to the high physical demands of sports [6,11,12,13]. These injuries affect the athlete’s immediate health and pose long-term cognitive, psychological, and physical challenges [14,15]. In this context, neurorehabilitation is crucial in enhancing motor recovery, maintaining physical performance, and ensuring a safe and timely return to play [8,16,17]. Moreover, the societal and economic burden of prolonged recovery or incomplete rehabilitation for injured athletes highlights the urgency of advancing neurorehabilitation methods tailored to the specific needs of this population [18,19,20,21,22].

Neurorehabilitation aims to enhance and restore functional ability and quality of life in individuals with physical impairments or disabilities that affect the nervous system [23,24,25]. In athletes, the focus of neurorehabilitation is not only on recovery but also on optimizing performance and aiding recovery from injury [26,27]. Physical therapy plays an indispensable role in this process, using different types of physical therapy modalities that harness neural plasticity for enhancing motor recovery post-neurological injuries [28]. These neurobiologically informed therapies leverage the behavioral and neural signals driving neural plasticity [29]. Physical therapy modalities include exercise, partial weight-based therapy, constraint-induced movement therapy, neurodevelopmental treatment, neurostimulation, transcranial magnetic stimulation, biofeedback, virtual reality therapy, and aquatic therapy [28,30]. These techniques have been incorporated into rehabilitation programs to facilitate recovery, improve motor function, and ensure a safe return to sport [31]. Recently, the integration of advanced technologies such as robotic-assisted therapy, virtual reality (VR) [32,33,34], and brain–computer interfaces (BCIs) [35,36,37] has begun to transform neurorehabilitation. These technologies offer novel opportunities for personalized rehabilitation, enhancing motor recovery, cognitive function, and overall therapeutic outcomes. However, the application and efficacy of these technologies in sports-specific neurorehabilitation has not been explored, presenting a critical area for future research.

Despite the significant advancements in neurorehabilitation, there is a notable gap in studies investigating the application of these therapies to sports. While general neurorehabilitation strategies are well-researched, few studies have focused on the demands of athletes, such as the need for early recovery and reaching peak physical performance as soon as possible. This bibliometric analysis seeks to address these gaps by focusing on how physical therapy interventions can be optimized for athletes in neurorehabilitation.

Bibliometric analysis, the quantitative analysis of academic publications (number of publications produced by a research unit, like a university or a researcher) and their impact (who/what is cited and how often) [38], has become an increasingly important tool in the field of physical therapy, providing valuable insights into the trends, impacts, and dynamics of research within the profession [39,40,41]. Moreover, bibliometric studies have been instrumental in tracking the evolution of thematic structures in physical therapy research over time [42,43,44].

Similarly, Tilson et al. [45] performed a bibliometric analysis of statistical terms in American Physical Therapy Association journals to guide curriculum development. Simon et al. [46] explored the scientific trends and content of articles published in the *Journal of Manual & Manipulative Therapy* between 1993 and 2012. Martínez-Fuentes et al. [47] conducted a co-citation analysis of three internationally recognized journals—*Physical Therapy*, *Physiotherapy*, and the *Australian Journal of Physiotherapy*—to map out the intellectual foundations of the physical therapy discipline. Wiles et al. [39] analyzed the research output of the *Physical Therapy Journal* from 1945 to 2010, while Coronado et al. [40] performed a bibliometric study of articles published in the same journal between 1980 and 2009.

Furthermore, bibliometric analyses have been applied to specific areas within physical therapy, such as the settings and monitoring of mechanical ventilation during physical therapy in critically ill patients [48], virtual reality-aided therapy [49], and physical activity therapy for diabetes [50], and focused on productivity and keyword trends in articles related to physical therapy and aging, offering valuable insights into research directions in this sub-field [51].

Despite the growing interest in neurorehabilitation, there is a notable lack of bibliometric analyses in the international literature regarding the application of physical therapy in athletes’ neurorehabilitation. To address this gap, we conducted a bibliometric analysis, followed by a narrative review of the key topics identified.

The primary aim of this study is to identify the key research trends, thematic clusters, and collaborative networks in physical therapy for sports neurorehabilitation. By applying advanced bibliometric methods, we aim to provide the international scientific community with a comprehensive understanding of the existing literature and lay the foundation for future research. The results of this research will not only guide the development of more effective rehabilitation strategies but also have a direct impact on clinical practice. These strategies could potentially reduce recovery times and enhance overall performance in athletes with neurological injuries, thereby improving the quality of care in sports neurorehabilitation.

## 2. Materials and Methods

### 2.1. Data Sources and Search Methods

The documents selected in this study were retrieved from the Scopus database (https://www.scopus.com/) on 26 July 2024. Scopus is one of the largest databases of curated abstracts and citations, indexing content across various disciplines [52], making it a valuable source for bibliometric data analysis [53]. Using the advanced search function, the search string included compound keywords, using the BOOLEAN expression (“physical therapy” OR “physiotherapy” OR “rehabilitation”) AND (“neurorehabilitation” OR “neurological rehabilitation” OR “neurorehabilitation”) AND (“athletes” OR “sports” OR “players”). Papers containing any of these terms in their titles, abstracts, or keywords were considered. From the initial search, 175 documents were identified. After reading the titles and abstracts, 72 documents were deemed unsuitable since they did not concern the application of physical therapy in sports neurorehabilitation. The first two authors systematically reviewed and reached a consensus on the eligibility of the papers. All types of documents (research articles, review articles, books, editorials, etc.) written in any language were accepted provided their topic was related to physical therapy in athletes’ neurorehabilitation.

### 2.2. Data Analysis

The CSV file obtained from Scopus was imported into VOSviewer (version 1.6.11) for bibliometric analysis. VOSviewer is a free software for creating, visualizing, and exploring bibliometric networks, which is particularly useful for visualizing the thematic structure of scientific disciplines [54]. Furthermore, after converting the file to an Excel (xls) format Microsoft Excel (version Office 365), Microsoft Corporation, Redmond, WA, USA, it was imported into Microsoft Power BI (Microsoft Power BI (version Office 365), Microsoft Corporation, Redmond, WA, USA) to visualize the documents per year. Power BI was also used to create a geographic map depicting the countries that participated in the writing of the documents. For the bibliometric analysis, both performance analysis and scientific mapping were conducted. Scientific mapping methods also include clustering techniques.

### 2.3. Performance Analysis

The performance analysis involved (a) calculating the number of documents per year, (b) ranking the sources and the countries with the most documents, and (c) ranking the authors with the most citations.

### 2.4. Scientific Mapping

The scientific mapping techniques used were as follows:

(a) Co-authorship analysis: countries were the unit of analysis, examining collaborations between countries based on the documents they have co-authored.
(b) Bibliographic coupling: sources were the unit of analysis, examining the extent to which two or more sources cite common references.
(c) Co-citation analysis: authors were the unit of analysis, analyzing the frequency with which two or more authors are cited together in other documents.
(d) Co-occurrence analysis: “all keywords” were the unit of analysis, examining the frequency with which two or more keywords appear together in the same documents. “All keywords” include keywords from the title or abstract and not just those defined by the author as “keywords”.

### 2.5. Narrative Review

Based on the clusters that emerged from the co-occurrence analysis, the narrative review was then carried out. In particular, each cluster was given a name by the authors based on the items (keywords) it included. The name of each cluster was the title for each sub-section of the narrative review.

## 3. Bibliometric Analysis Results

### 3.1. Performance Analysis

**Figure 1:**
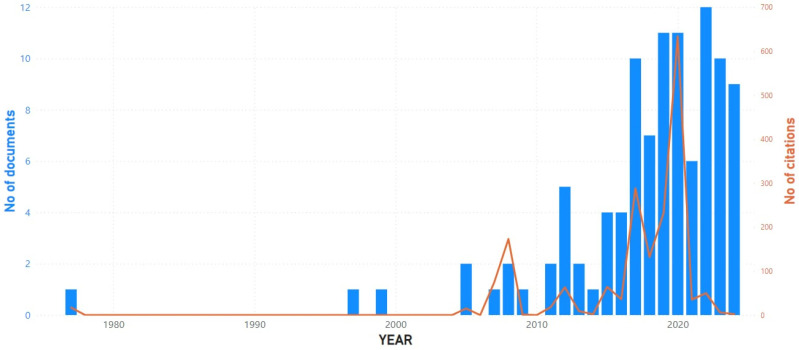
Number of documents and citations per year.

A total of 103 articles were considered suitable for inclusion in the bibliometric analysis. Figure 1 shows the number of documents and citations per year. Until 2011, publications were very rare, but from 2012 onwards, they started to become more frequent. Starting in 2017, there has been a nearly steady trend in a double-digit number of publications each year.

Table 1 shows the number of documents and citations and impact factors (IFs) from the 17 sources that have at least two relevant documents. The ranking order is primarily based on the number of documents. The *Journal of Head Trauma Rehabilitation* ranks first in the number of documents, while the *Journal of Neuroengineering and Rehabilitation* ranks first in citations.

**Figure 2:**
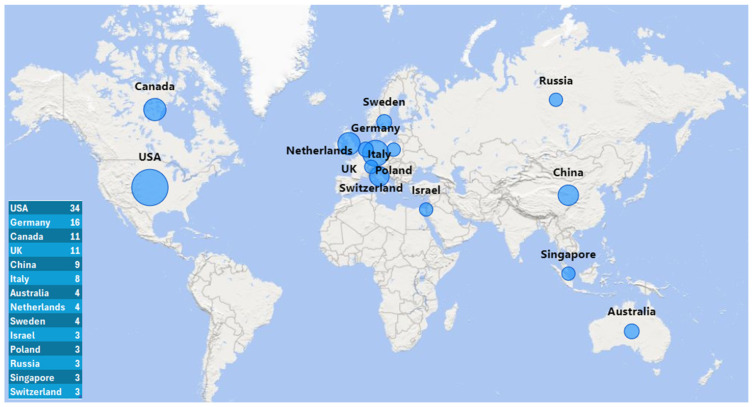
Geographical map of the countries with at least 3 documents.

The geographical map in Figure 2 illustrates the distribution of documents across different countries, with the size of the bubbles representing the number of documents published by each country. Only countries with at least three documents are included in the map. The USA is the most prolific, with 34 documents, followed by Germany, with 16 documents.

Table 2 presents the ranking of authors based on citations, highlighting the 16 authors who have contributed to at least two relevant documents. Dharm-Datta, Shreshth and Ellis, Henrietta, who co-authored two documents [55,56], are ranked first. The only authors who had two documents as first authors were Baur K. and Braun S., while the rest had one each. This table also shows the total number of citations for each author, regardless of the article’s topic.

### 3.2. Science Mapping

**Figure 3:**
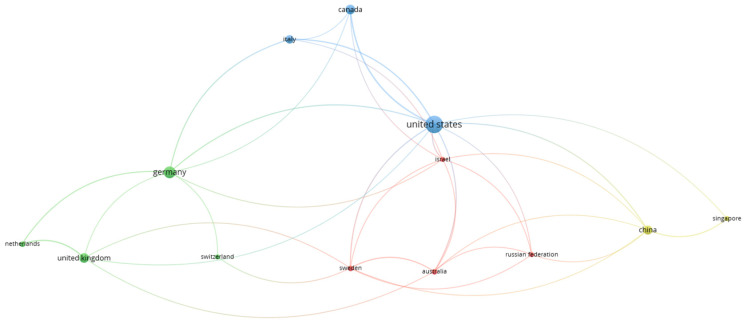
Co-authorship network among countries. Different colors signify clusters of countries that tend to collaborate more frequently with each other.

Figure 3 illustrates the results of the co-authorship analysis, with countries as the unit of analysis. This analysis reveals the networks of cooperation between countries based on jointly authored scientific papers. The figure highlights that the United States and Germany are the most central countries in the network, evidenced by their largest bubbles and the most connections to other countries. This indicates that these countries have a strong presence in the field of physical therapy and neurorehabilitation of athletes, as well as extensive collaborations with other nations.

**Figure 4:**
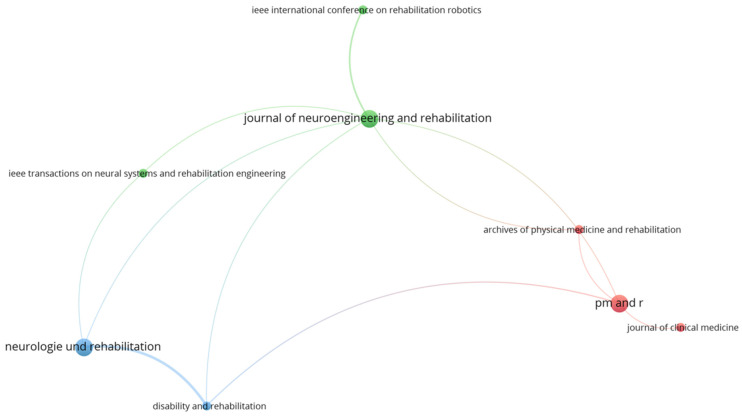
Bibliographic coupling among key journals. The colors differentiate between clusters of journals that are more closely connected in terms of shared references.

Figure 4 illustrates the results of bibliographic coupling with the sources as the unit of analysis. This analysis examines the extent to which two or more sources cite common references, thus determining the connection between them in terms of subject matter and their scientific basis. In the figure, the *Journal of Neuroengineering and Rehabilitation* appears to be the central hub, linked to several other important sources. This suggests that this journal is a central source of knowledge and holds a substantial influence in the field of neurorehabilitation and neurorehabilitation engineering.

**Figure 5:**
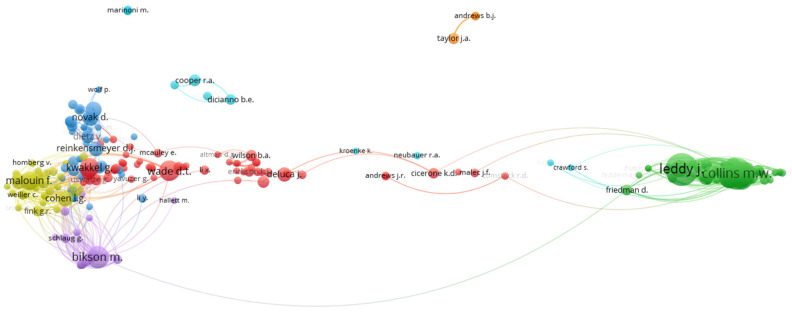
Co-citation network among authors. Each color signifies a distinct group of researchers whose work is closely interconnected based on shared citations.

Figure 5 presents the results of the co-citations analysis, with authors as the unit of analysis. This analysis examines how often two or more authors are cited together in the bibliographies of other scholarly works, highlighting which authors are central and influential in the field and their relationships to one another. The arrangement of the nodes representing different authors indicates groups or communities of authors who are connected through their citations. The colors correspond to different clusters, meaning groups of authors who are frequently co-cited. The sizes of the circles represent the authors’ relative citation frequency, with larger circles corresponding to more central and influential authors in the field. From the figure, it is evident that authors such as Leddy J. and Collins M.W. form a large and central cluster on the right, while others, such as Bikson M., Kwakke L., and Malouin F., form other significant co-citation groups on the left side of the network.

**Figure 6:**
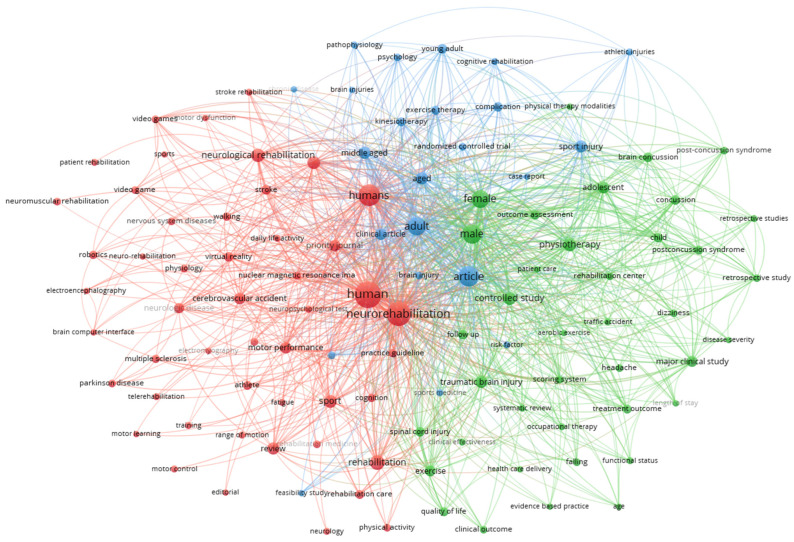
Co-occurrence network of keywords. Each color signifies a distinct thematic group, indicating closely related research topics or areas.

Figure 6 presents the results from the co-occurrence analysis of “all keywords”. This analysis reveals thematic relationships between terms that frequently appear together in the same documents, highlighting the main research directions and areas of interest in the field of physical therapy and neurorehabilitation of athletes.

Table 3 shows all the items within each cluster. Based on the items, each cluster was named, and we proceeded with the narrative review of the three identified topics. The identification of the “Athlete-Centered Neurorehabilitation Techniques” cluster suggests the importance of tailoring rehabilitation strategies to individual athletes’ needs, leveraging advanced technologies to enhance recovery. This highlights a growing trend towards personalized care in sports medicine. Additionally, the “Clinical Approaches and Outcomes in Neurorehabilitation” cluster underscores the importance of evidence-based practice in optimizing intervention strategies to improve patient outcomes.

Moreover, the “Specialized Interventions in Sports Medicine and Neurorehabilitation” cluster accentuates the integration of specialized techniques and multidisciplinary approaches to address the complex needs of athletes recovering from neurological injuries. This cluster emphasizes the necessity of tailored interventions that encompass physical rehabilitation and psychological and cognitive elements crucial for holistic recovery. Future research in this area should focus on assessing the efficacy of these specialized interventions across various sports and injury types, considering their long-term impact on athletes’ performance and well-being.

## 4. Narrative Review

### 4.1. Clinical Approaches and Outcomes in Neurorehabilitation

In recent years, there has been growing interest in understanding the role of neuroplasticity in neurorehabilitation, which describes the brain’s ability to reorganize and adapt in response to experience and practice and form the basis for many neurorehabilitation approaches [57]. Evidence suggests the brain can reorganize and adapt after injury, improving function and recovery [58,59]. Preclinical research has shed invaluable light on the physiological and molecular mechanisms underlying this dynamic neuroplastic capacity, revealing how the brain can fundamentally alter the properties of its neural circuits in the aftermath of injury [60,61,62]. The literature has explored various clinical approaches in neurorehabilitation, in which neuroplasticity plays a crucial role in recovery [63]. One prominent example of the application of neurorehabilitation is in the context of motor recovery after stroke. Recent randomized clinical trials have emphasized the requirement for intense progressive rehabilitation programs to optimally enhance recovery [58]. These programs often incorporate techniques such as impairment-oriented training, constraint-induced movement therapy, electromyogram-triggered neuromuscular stimulation, and robotic interactive therapies [57].

In the realm of neurorehabilitation, integrating technology such as virtual reality, intelligent robotics, and brain–computer interfaces has opened up new possibilities for enhancing therapy outcomes [64,65,66]. These technologies offer opportunities for remote therapy delivery, personalized rehabilitation, and innovative approaches to motor-cognitive training [64,65,66].

Holistic neurorehabilitation includes therapies that focus on various aspects of functioning, such as physical, cognitive, language, emotional, and interpersonal functioning, including training in compensatory strategies [67]. Comprehensive rehabilitation planning for patients with brain injuries caused by lightning and electrical trauma involves a multidisciplinary team approach, which addresses both physical and cognitive deficits [68]. Similarly, the incorporation of neuropsychology services within a multidisciplinary concussion clinic has demonstrated significant benefits, with early referral for specialized care leading to improved physical and cognitive outcomes [69,70].

In the context of neurorehabilitation for acquired brain injuries, the interplay between psychosocial functioning and motor and cognitive recovery has been emphasized, indicating that psychosocial factors, particularly participation, play a crucial role in driving overall recovery outcomes [71]. This underscores the importance of considering not only the physical aspects of rehabilitation but also the psychological and social dimensions to achieve comprehensive and sustainable improvements in patient well-being.

Moreover, the impact of comorbidities on neurorehabilitation outcomes, particularly in conditions like multiple sclerosis, has been a subject of investigation, highlighting the need to address additional health issues that may influence the effectiveness of rehabilitation interventions [72]. Understanding how comorbidities interact with the neurorehabilitation process is essential for optimizing treatment strategies and improving overall outcomes for individuals with complex health profiles.

In pediatric neurorehabilitation, applying the International Classification of Functioning, Disability, and Health (ICF) as a clinical reasoning tool has been proposed to guide therapists in developing comprehensive rehabilitation strategies tailored to the specific needs of children with neurological conditions [73]. By adopting a structured approach that considers the functional, social, and environmental factors impacting a child’s rehabilitation, clinicians can better address the diverse challenges faced by pediatric neurorehabilitation patients.

Additionally, research into the influence of personality on neurorehabilitation outcomes has shown that individual traits and psychological profiles significantly impact the recovery process following neurological injuries like stroke or brain tumors [74]. The application of motor imagery and mental practice in stroke rehabilitation highlights the importance of personalized interventions tailored to the specific capabilities of each patient [75]. Advanced imaging techniques such as functional magnetic resonance imaging (fMRI) facilitate detailed assessments of cerebral activation changes post-treatment, offering valuable insights into the efficacy of behavioral interventions [69,70]. This highlights the importance of personalized approaches in neurorehabilitation that take into account each patient’s unique psychological makeup to optimize treatment outcomes and enhance overall well-being.

Furthermore, integrating noninvasive brain stimulation techniques with traditional neurorehabilitation approaches have been proposed to maximize functional recovery by leveraging the brain’s plasticity and adaptive mechanisms [76]. Combining innovative technologies like transcranial direct current stimulation (tDCS) has shown promise in enhancing motor cortex modulation, potentially improving cognitive functions and motor learning in both neurological diseases and sports performance [77,78].

The effectiveness of neurorehabilitation is typically assessed through various key outcomes. Functional independence, a patient’s ability to perform activities of daily living autonomously, is commonly evaluated using tools like the Functional Independence Measure (FIM) or the Barthel Index [79,80,81]. Motor recovery, particularly in conditions such as stroke and spinal cord injuries, focuses on restoring movement and coordination and is typically assessed through specific motor scales [82]. Cognitive improvement, crucial for daily functioning, involves enhancements in memory, attention, and problem-solving abilities and is measured using neuropsychological tests [83,84]. Psychosocial well-being, which encompasses reduced symptoms of depression and anxiety, improved social participation, and an overall enhancement in quality of life, is another critical outcome of neurorehabilitation [85]. Additionally, reducing disability severity contributes to better long-term outcomes and decreased healthcare costs [86].

Neurorehabilitation is a dynamic and multidimensional field that continues to evolve with advancements in technology, personalized medicine, and a growing understanding of the complex interplay between biological, psychological, and social factors in rehabilitation outcomes. By integrating evidence-based practices, innovative technologies, and personalized approaches, clinicians can optimize the effectiveness of neurorehabilitation interventions and improve the quality of life for individuals with neurological dysfunctions.

### 4.2. Athlete-Centered Neurorehabilitation Techniques

Athlete-Centered Neurorehabilitation Techniques have gained significant attention in recent years as a means to enhance performance, prevent sports injuries, and facilitate the return of athletes to their respective sports. Integrating technology and personalized approaches in sports rehabilitation has shown promising results, with techniques such as virtual reality/augmented reality, motion tracking, biomechanical analysis, and neurostimulation playing pivotal roles in optimizing athletes’ recovery [87,88,89,90]. A study that introduced guided activity-based gaming for stroke rehabilitation demonstrated significant improvements in motor functions, as measured by the Wolf Motor Function Test and the Fugl-Meyer Assessment [91]. These innovative approaches highlight the potential of integrating gaming into neurorehabilitation protocols for athletes, providing both motivation and measurable outcomes and ensuring a holistic approach to athlete well-being [91,92,93]. Similarly, the use of virtual reality in stroke has shown promise in addressing sensory-motor impairments and facilitating sensory-motor reorganization in patients post-stroke [68,93].

Incorporating advanced technologies such as robotics and brain–computer interfaces (BCIs) has revolutionized neurorehabilitation. A review of contemporary motor rehabilitation approaches emphasized the role of robotics and BCIs in optimizing human motion performance, enabling precise control over therapeutic exercises and ensuring that the interventions are both effective and adaptable to the athletes’ needs [92,94,95]. The development of a computer game for Parkinson’s disease patients using Microsoft Kinect further demonstrated the feasibility of such systems for home-based rehabilitation, allowing athletes to continue their recovery independently while promoting sustained improvements in motor functions and overall quality of life [92].

Moreover, randomized controlled trials evaluating the impact of exercise therapy programs based on sports in individuals with acquired brain injury have highlighted the potential benefits of integrating sports-specific interventions into neurorehabilitation protocols [96,97,98]. Vestibular rehabilitation therapy for adolescents with post-concussion syndrome has been shown to significantly reduce symptoms of dizziness, unsteadiness, and imbalance, which are common in athletes post-concussion. Early evaluation and individualized vestibular rehabilitation therapy programs can expedite recovery and minimize time away from sports [99,100]. Similarly, transcranial direct current stimulation (tDCS) has shown promise in enhancing motor cortex modulation, with potential benefits in improving cognitive functions and motor learning in both neurological diseases and sports performance [75,77].

Furthermore, incorporating mindfulness, acceptance, and self-compassion practices in rehabilitation protocols for injured athletes has been shown to enhance commitment therapy and foster a more athlete-centered focus during the recovery process [101,102,103]. Integrating mental practice and motor imagery in neurorehabilitation plays a crucial role in enhancing motor recovery, with tailored interventions based on individual patient capabilities demonstrating significant benefits [75,104]. Additionally, the application of movement imagery in neurorehabilitation has been shown to enhance physical performance and self-determination in patients, effectively adapting a technique from sports science to rehabilitation settings [105,106].

Overall, athlete-centered neurorehabilitation techniques are critical in optimizing recovery, enhancing performance, and promoting holistic well-being in the athletic population. By tailoring rehabilitation strategies, incorporating advanced technologies and tailored exercise strategies, and addressing both the physical and psychological aspects of recovery, practitioners can effectively support athletes on their journey to returning to sport and help them achieve their performance goals. Continued research and development in this field are likely to yield even more effective interventions, further enhancing the recovery and performance of athletes.

### 4.3. Specialized Interventions in Sports Medicine and Neurorehabilitation

Specialized Interventions in Sports Medicine and Neurorehabilitation encompass various techniques and technologies designed to enhance recovery and performance in individuals with neurological conditions or sports-related injuries [6,8,10]. Injury rehabilitation is a critical component of sports medicine, and advancements in our understanding of the physical, psychological, mental, and emotional aspects of the recovery process continue to evolve [8,96,103]. The integration of a comprehensive and qualified sports medicine team should be considered the standard of care to which all athletes are entitled.

Technology-based interventions have also gained prominence in neurorehabilitation, offering opportunities to enhance recovery outcomes [107,108,109]. Virtual reality rehabilitation has been explored as a tool for behavior change in neurologic populations, showing the potential to improve outcomes and quality of life for individuals with nervous system injuries [108]. Health-related multiplayer games have been found to improve motivation and performance in patients, with virtual reality and robotic assistance enabling highly individualized and engaging therapy sessions [92,95]. Additionally, wearable technologies, such as IMU-based wearables, have shown promise in sports medicine by providing physical therapists and rehabilitators with valuable data for monitoring and optimizing rehabilitation progress [110].

Cognitive and motor rehabilitation constitute essential elements of neurorehabilitation for athletes. The examination of treatments and rehabilitation approaches for traumatic brain injuries emphasizes the importance of a well-coordinated, interdisciplinary rehabilitation process, particularly for athletes recovering from concussions sustained in sports [70,111]. Additionally, the impact of neural plasticity on recovery after a stroke, as observed in athletes and professional musicians, highlights the potential of personalized rehabilitation strategies to boost neural plasticity and support functional recovery [104,106].

Incorporating mental practice and motor imagery in neurorehabilitation is critical for enhancing motor recovery, as tailored interventions based on individual patient capabilities have shown significant benefits by being customized to address each patient’s specific needs and limitations [75,104]. Advanced neuroimaging techniques, such as functional magnetic resonance imaging (fMRI), facilitate the detailed evaluation of changes in cerebral activation before and after treatment, providing valuable insights into the effectiveness of behavioral interventions elucidating the underlying neural mechanisms that contribute to improved motor function and recovery [69,105]. Sensory interventions, like electrical stimulation, have been explored as a way to enhance touch sensation and sensory retraining in neurorehabilitation [112]. Despite the prevalence of somatosensory impairments, further research is still needed to elucidate the effectiveness of interventions like sensory electrical stimulation in improving post-injury sensory function and processing [112]. Moreover, the ecological validity of interventions in pediatric neurorehabilitation has been emphasized to provide a comprehensive understanding of an individual’s functionality in real-life situations, underscoring the importance of considering daily life contexts in rehabilitation approaches [113,114].

Specialized Interventions in Sports Medicine and Neurorehabilitation provide diverse approaches to enhancing recovery outcomes for athletes recovering from neurological injuries. By leveraging technological advancements and applying the principles of motor learning and brain plasticity, practitioners can tailor interventions to meet each patient’s unique needs. Continued research and development in this field will likely result in even more effective interventions, further enhancing athletic recovery and performance.

## 5. Discussion

This study aimed to provide comprehensive knowledge of the scientific literature in the field of physical therapy in sports neurorehabilitation. This was achieved through a bibliometric analysis, where performance analysis, science mapping, and clustering techniques were employed, as well as through a narrative review of the key topics that emerged from the clustering technique of a co-occurrence analysis of the documents’ keywords.

The sharp increase in related publications, particularly from 2017 onwards, can be explained by technological advancements such as intelligent robotics, virtual reality, and brain–computer interfaces, which have been integrated into physical therapy and neurorehabilitation [64,65,66]. This, combined with the fact that professional teams aim to reduce recovery time [115], has heightened research interest in this specific field.

Noteworthy is the observation in Table 1, where it is evident that the journal *Neurologie und Rehabilitation*, despite being one of the journals with the most relevant documents, has very few citations. This can be explained by the fact that these articles are written in German rather than English. Scientific papers published in English typically have a broader reach (more citations) and are more widely recognized by the international scientific community, which primarily uses English as the lingua franca [116].

The co-authorship analysis revealed strong collaboration between the USA, Canada, Germany, and the United Kingdom. International collaboration in research is essential for advancing the field, as it allows for sharing diverse expertise and enhancing the impact and quality of research outputs. Studies have shown that international collaborative papers tend to have a higher impact and broader reach than non-international collaborative papers [117]. Major international healthcare organizations and research funding bodies consider international collaborative research as an indicator of research quality [118]. This suggests that patients receive care that complies with current research evidence guidelines (World Confederation for Physical Therapy-European Region, 2015) and improves clinical practice [119]. Furthermore, these collaborations, along with the general partnerships among all countries, demonstrate the need for international cooperation in research overall [120,121], specifically in addressing modern challenges and developing new techniques in neurorehabilitation. This knowledge exchange accelerates the development of cutting-edge therapies and ensures that best practices worldwide are integrated into sports neurorehabilitation protocols, enhancing recovery outcomes for athletes globally. International collaborations enhance access to broader funding, resources, and cutting-edge technologies. Additionally, collaborations facilitate the creation of protocols that can be modified for application across diverse cultures, religions, populations, and healthcare systems [122]. This is especially advantageous for elite athletes who may train or compete internationally, as it ensures they receive consistent, high-quality care regardless of location.

Additionally, from the bibliographic coupling with the sources as the unit of analysis, the different-colored nodes indicate the formation of thematic clusters, that is, groups of journals that share common references and likely focus on similar thematic areas. For example, the green journals seem to relate to technology and engineering in rehabilitation, the red ones focus on clinical medicine and rehabilitation, and the blue ones appear to involve journals primarily concerned with physical therapy or more general aspects of rehabilitation. The collaboration and contribution of different sub-fields allow for a more holistic and effective approach to sports neurorehabilitation. This multidimensional nature is essential for developing innovative and efficient rehabilitation strategies incorporating best practices from each field. Therefore, it is natural for journals from various sub-fields to be included, reflecting the complexity and interdisciplinary nature of research in this area.

To the best of our knowledge, this study represents the most comprehensive delineation and identification of topics in sports physical therapy in neurorehabilitation. It is distinguished by the large sample of identified articles and the advanced methodology employed in delineating and analyzing the publications. More importantly, no other bibliometric review exists in sports neurorehabilitation. This study employs advanced bibliometric methods, including performance analysis, co-authorship analysis, bibliographic coupling, and co-occurrence analysis. These techniques allow for a more structured and detailed mapping of the key topics, influential authors, and research trends in sports physical therapy for neurorehabilitation. Previous reviews, while valuable, primarily relied on systematic or literature reviews, which may not capture the same depth and breadth of research dynamics and collaborative networks.

Physical therapy is a field that has been extensively studied through various research articles. Carballo-Costa et al. [44] conducted a bibliometric analysis from 2000 to 2018, focusing on the thematic structure of physical therapy and introducing a novel publication-level classification approach. This study stands out for its advanced methodology in delineating the field. Similarly, Moral-Munoz et al. [42] provided an overview of the thematic evolution of physical therapy research from 1951 to 2013, analyzing publications from the Web of Science and Scopus databases. These studies contribute significantly to understanding the development and trends within the field of physical therapy. Our bibliometric analysis spans a longer timeframe and includes more recent publications, ensuring that the latest advancements in rehabilitation technologies and athlete-centered techniques are incorporated. The bibliometric analyses mentioned above had a completely different scope, not focusing on sports neurorehabilitation. Additionally, this study goes beyond simple content analysis by identifying three key thematic clusters: Athlete-Centered Neurorehabilitation Techniques, Clinical Approaches and Outcomes in Neurorehabilitation, and Specialized Interventions in Sports Medicine and Neurorehabilitation. These clusters represent distinct areas of research focus within sports neurorehabilitation.

Despite the valuable insights offered by this study, it has its limitations. The first and most significant limitation is that the analysis was based exclusively on documents retrieved from the Scopus database., which has a different coverage from PubMed or Web of Science. Also, specialized databases like the Physiotherapy Evidence-Based Database (PEDro), which is recognized for its comprehensive indexing of studies related to the effects of physical therapy interventions, were excluded from the analysis. However, including multiple databases is uncommon in bibliometric analyses due to the complexity of data harmonization and the potential for duplication. Nevertheless, we acknowledge this point and will consider this in future research. Studies have shown that the quality of reports of randomized controlled trials (RCTs) varies across physiotherapy subdisciplines, with PEDro being identified as the most complete index for RCT reports in physiotherapy interventions [123]. Additionally, publication bias could skew the results, as studies with positive findings are more likely to be published [124]. Using only keywords and journals for field delineation is considered a less precise approach with lower recall potential [44,125,126]. This method may not capture the full scope of a field due to its precision and recall limitations. Lastly, given the interdisciplinary nature of neurorehabilitation in athletes, a broad search strategy was necessary to ensure a comprehensive capture of relevant studies across fields such as sports medicine, neurobiology, and rehabilitation technologies. By using a wide-ranging search string, we aimed to avoid overlooking important studies that may not have been categorized under traditional physiotherapy terms but are highly relevant to the neurorehabilitation of athletes. This approach aligns with best practices in bibliometric research, particularly in fields where multiple disciplines converge. While this may have led to the initial inclusion of some peripheral studies, a systematic review process was employed to ensure that only those directly relevant to the application of physical therapy in sports neurorehabilitation were included in the analysis.

Future research in physical therapy and neurorehabilitation should prioritize several key areas based on the current literature. A notable limitation of this study is the exclusive reliance on the Scopus database, which, while comprehensive, may not contain all relevant literature, especially from specialized databases like PEDro. Databases such as Scopus, PubMed, and Web of Science include a broad spectrum of healthcare research, whereas PEDro specifically indexes evidence related to the effects of physical therapy interventions. This could result in the exclusion of key studies, potentially affecting the thematic clusters identified in our analysis. Future research should compare data from our findings to PEDro to uncover additional insights, providing a more complete understanding of the research landscape. Additionally, expanding the geographical scope of research, especially to include underrepresented regions, would offer a global perspective on physical therapy practices and outcomes.

## 6. Conclusions

This study provides the most extensive delineation and identification of thematic areas in physical therapy and neurorehabilitation for athletes, employing advanced bibliometric techniques. The notable growth in publications since 2017 highlights the growing trend towards utilizing cutting-edge technologies, such as robotics and virtual reality. The analysis has highlighted the prominent contributions from the USA and Germany, along with influential journals and authors. Three primary thematic areas were identified: Athlete-Centered Neurorehabilitation Techniques, Clinical Approaches and Outcomes in Neurorehabilitation, and Specialized Interventions in Sports Medicine and Neurorehabilitation. These thematic clusters emphasize the interdisciplinary and dynamic nature of research, emphasizing the personalized, technology-driven interventions used to optimize athlete recovery. However, the exclusive use of the Scopus database poses a limitation, potentially excluding relevant literature from specialized databases like PEDro and thereby limiting the scope.

Future research should focus on the long-term effectiveness of personalized interventions and integrating Artificial Intelligence (AI) and wearable technologies. At the same time, studies are encouraged to expand the geographical scope to include underrepresented regions, providing a more global perspective on physical therapy and neurorehabilitation practices, thereby enriching the knowledge base of the field. For clinicians, implementing technology-driven, individualized rehabilitation approaches can significantly improve the recovery and performance of athletes. It is essential to incorporate specialized interventions, such as vestibular rehabilitation, to address complex neurological injuries effectively. The field can progress further by tackling these deficiencies, leading to enhanced athlete outcomes.

## Figures and Tables

**Table 1:** Number of documents and citations and impact factors (IFs) for the sources.

Source	Documents	Citations	IF
*Journal of Head Trauma Rehabilitation*	5	56	2.26
*Journal of Neuroengineering and Rehabilitation*	4	205	5.61
*Pm and R*	4	153	2.03
*Frontiers in Neurology*	4	126	2.80
*Neurologie und Rehabilitation*	4	4	0.23
*Brain Injury*	3	46	1.55
*Neurorehabilitation*	3	35	1.68
*IEEE Transactions on Neural Systems and Rehabilitation Engineering*	2	75	5.44
*Disability and Rehabilitation*	2	34	2.65
*IEEE International Conference on Rehabilitation Robotics*	2	25	0.86
*Archives of Physical Medicine and Rehabilitation*	2	23	3.13
*Medicine and Science in Sports and Exercise*	2	13	3.70
*Rehabilitation*	2	13	0.75
*Current Sports Medicine Reports*	2	4	1.28
*Neurology*	2	4	3.79
*BMJ Open*	2	3	2.53
*Journal of Clinical Medicine*	2	3	3.07

**Table 2:** Ranking of authors based on citations.

Author	Documents	Citations	Total Citations per Author, Regardless of Article Topic
Dharm-Datta, Shreshth	2	436	654
Ellis, Henrietta	2	436	451
Riener, Robert	3	136	15,953
Sunnerhagen, Katharina S.	2	100	14,736
Baur, Kilian	2	61	229
Duarte, Jaime E.	2	61	464
Wolf, Peter	2	61	10,942
Gagnon, Isabelle	3	60	5192
Friedman, Debbie	2	52	14,642
Grilli, Lisa	2	52	535
Iosa, Marco	2	32	6416
Morone, Giovanni	2	32	5860
Paolucci, Stefano	2	32	10,282
Wade, Derick T.	2	29	34,641
Leddy, John J.	2	20	12,102
Willer, Barry S.	2	20	6933

**Table 3:** The items included in each cluster.

Cluster 1 (Red): Athlete-Centered Neurorehabilitation Techniques	Cluster 2 (Green): Clinical Approaches and Outcomes in Neurorehabilitation	Cluster 3 (Blue): Specialized Interventions in Sports Medicine and Neurorehabilitation
Athlete, brain–computer interface, cerebrovascular accident, cognition, daily life activity, electromyography, fatigue, gait, human, motor control, motor dysfunction, motor learning, motor performance, multiple sclerosis, nervous system diseases, neurorehabilitation, neurologic disease neurological rehabilitation, neurology, neuromuscular rehabilitation, neuropsychological test, Parkinson’s disease, physical activity, priority journal, procedures, range of motion, rehabilitation care, rehabilitation medicine, robotics, sport, sportsstroke, stroke rehabilitation, telerehabilitation, video game, video games, virtual reality, and walking	Adolescent, aerobic exercise, age, brain concussion, child, clinical effectiveness, clinical outcome, concussion, controlled study, disease severity, dizziness, evidence-based practice, exercise, falling, female, follow up, functional status, headache, healthcare delivery, length of stay, major clinical study, male, occupational therapy, outcome assessment, patient care, Physical therapy modalities, physiotherapy, post-concussion syndrome, quality of life, rehabilitation center, retrospective studies, retrospective study, scoring system, spinal cord injury, systematic review, traffic accident, traumatic brain injury, and treatment outcome	Adult, aged, article, athletic injuries, brain injuries, brain injury, case report, chronic disease, clinical article, cognitive rehabilitation, complication, exercise therapy, feasibility study, kinesiotherapy, middle aged, pathophysiology, psychology, questionnaire, randomized controlled trial, risk factor, sport injury, sports medicine, and young adult
